# Characteristics of adolescents who visit the emergency department following suicide attempts: comparison study between adolescents and adults

**DOI:** 10.1186/s12888-019-2213-5

**Published:** 2019-07-26

**Authors:** Jinhee Lee, Yeon Sik Bang, Seongho Min, Joung-Sook Ahn, Hyun Kim, Yong-Sung Cha, In-Suk Park, Min-Hyuk Kim

**Affiliations:** 1 Department of Psychiatry, Yonsei University Wonju College of Medicine, 20 Ilsan-ro, Wonju, 26426 Republic of Korea; 2 Department of Emergency Medicine, Yonsei University Wonju College of Medicine, Wonju, Republic of Korea; 3 Yonsei Soul Psychiatric Clinic, Wonju, South Korea

## Abstract

**Background:**

The purpose of this study was to identify the demographic and clinical characteristics of suicide attempts in adolescents who visit the emergency department compared to those of adults.

**Methods:**

This study included 149 children under the age of 18, and 1427 people in the age of 19–65 who came to the emergency department with suicide attempt from 2009 to 2015. We compare sociodemographic, clinical, and suicide attempt-related characteristics through Chi-square test and logistic regression analysis to evaluate the difference between two groups.

**Results:**

In adolescents, suicide attempters had more number of previous suicide attempt history than adults. Adolescents used more non-lethal method such as poisoning of over the counter drugs and had about 5 times higher odds ratio in suicide attempts with analgesics. The motivation of suicide attempt among adolescents was more related with interpersonal problems but less with financial or illness-related problems. The intention of suicide attempt in adolescents was less serious and lethal compared to adults.

**Conclusion:**

Suicide attempts among adolescents had showed different from adults in method, motivation and intention. Considering the characteristics of suicide attempt among adolescent, it is necessary to keep close attention to adolescent’s suicide attempters and develop the customized intervention program to prevent the suicide attempt in this groups.

## Background

Suicide is one of the most important issues related to mental health, both personally and socially. Every year close to 800,000 people died from suicide, and suicide attempt rate are about 10 times higher than suicide rate [1, 2]. According to OECD’s survey results, in South Korea in the year 2013, 28.7 people per 100,000 population attempted suicide and recorded the 1st place with more than twice the suicide rate of other OECD countries [3, 4]. This serious suicide problem applies not only to adults but also to adolescents. The most common cause of death among teenagers in Korea in 2016 is suicide [5]. Unlike other OECD countries, the death rate of adolescents’ suicides in Korea has shown an increasing trend [6].

Suicide attempts are caused by complex factors such as genetic, environmental, cultural and psychiatric factors [7]. Adolescents’ suicide attempts differ from those of adults, because of their physical changes and their varied emotional experiences in their environment. According to previous studies, adolescents attempted suicide more impulsively than adults and opted for less fatal methods of attempts [8]. Suicide attempters among adolescents had fewer number of psychiatric diagnoses [9] and their mental disorders are easily less identified or diagnosed by professional [10]. Also they had fewer suicidal motivations due to economic problems and physical illnesses, and used more over the counter drug poisoning as a suicide attempt than adults [11].

Although previous studies have described risk factors for suicide and suicidal behavior in adolescents [12–14], very few studies have directly compared demographic and clinical features with adults. In addition, most of these studies were carried out with patients hospitalized after attempted suicide. Therefore, the study conducted on patients visiting the emergency department (ED) is very limited. Considering that the proportion of patients admitted to medical facilities due to attempted suicide is small, the results of previous researches are limited in representing the characteristics of the entire suicide attempt [15].

Since suicide attempt in adolescents is a risk factor for subsequent retries and suicide death, identifying the distinct characteristics of patients who visited an ED has clinical significance in establishing prevention strategies for adolescents’ suicides. We hypothesized that unique characteristics may have influenced suicidal behavior in adolescent group and identification of the characteristics of suicide attempters among adolescents will help establish efficient preventive strategies (i.e., specific target, points of access, methods, and intensity of care). In the context, the purpose of this study was to identify the demographic and clinical characteristics of suicide attempts in adolescents compared with those of adults who visited the ED.

## Methods

### Study subject

The subjects were under 65 years old, visited in ED in Wonju city, South Korea, from March 01, 2009 to December 31, 2015.

Wonju is a city with a population of 330,000 and approximately 10% of the population is engaged in agriculture. The ED is the only regional emergency medical center that covers the city, neighboring small towns and rural areas. Most of suicide attempters visit the ER for medical care.

For comparison, adolescents and adults were divided into two groups: adolescents between 12 and 18 years of age and adults between 19 and 65 years of age. Patients older than 65 years were defined as elderly and were excluded from this study, because they were thought to have characteristics distinguished from adults aged 65 and under [16]. For the suicide re-attempters, we used the data collected at the first ED visit during the study period.

The definition of suicide attempt was based on the WHO criteria [17], as a nonfatal self-directed potentially injurious behavior with any intent to die as a result of the behavior. In this study, we defined ‘suicide attempts’ as incidents when the patients and caregivers reported obvious intent. Only when the attempters were unable to provide reliable information due to mental confusion or severe physical damages, medical staff confirmed the injuries as possible suicide attempts using clear evidences (i.e., obvious circumstance, witness reports).

### Data collection and measurement

For patients who visited the ED, medical/surgical initial assessment and first aid for injuries were performed. Then psychiatric interviews were conducted by psychiatric residents and research assistants (i.e. trained nurse and social workers). When the attempters were unable to provide reliable information because of mental confusion or severe physical damage, only their caregiver was interviewed. When informant discrepancies arose between them, we relied on the attempters’ report, except when they were mentally confused. If emergency surgery is needed due to severe injury, authors confirmed the missing information via consult after hospitalization.

Factors related to suicide attempts were classified as socio-demographic, clinical, and suicide attempt-related characteristics.

#### Socio-demographic and clinical characteristics

Socio-demographic characteristics include age, gender, marital status, education level, income, religion. Clinical characteristics include medical illness, psychiatric illness history, psychiatric family history, family history of suicide, previous suicide attempt, current psychiatric treatment, and clinical psychiatric diagnosis. Based on the collected information, the clinician who interviewed patients and caregivers made an estimated-diagnosis by DSM - IV - TR, and two psychiatrists confirmed the diagnosis. The diagnoses were classified into a total of 10 disorder groups; anxiety disorders, somatoform disorder, depressive disorder, bipolar disorder, schizophrenia or other psychotic disorders, substance disorders, mental retardation / learning disorders / developmental disorder, dementia or organic mental disorder, adjustment disorder, personality disorder. If more than 2 diagnoses were made, the diagnosis that had the greatest effect on suicide attempts was set as 1st diagnosis.

#### Suicide attempt-related characteristics

Suicide attempt-related characteristics include method, motivation, intention to die, attempts with suicidal plan, sustained suicidal ideation, medical lethality, use of alcohol before the attempt, joint suicide attempts. The method of suicide attempt was divided into 6 groups. (i.e. poisoning, asphyxia by gas, hanging, injury by sharp object, jumping and drowning, others). Also, poisoning was divided into analgesics, psychiatric medication, herbicide and pesticide, and other medications. Multiple selections were allowed for the type of medication used. The motivation for suicide attempt was divided into 8 groups (i.e. psychiatric, interpersonal, job-related, economic, illness-related, loss, abuse-related, legal problem). Multiple selections were allowed. The intention to die was classified as one of ‘some or little’, ‘certain’ in consideration of self-report on suicidal intent, objective circumstances, daily life, and methods. The attempts with suicidal plan means attempters had made a specific plan to suicide attempt and suicide attempt had been the consequence of the plan, and the sustained suicidal ideation means the suicide attempters had suicidal ideation consistently after the suicide attempt which have brought the attempters to the hospital. Medical lethality was divided into ‘mild to moderate (no damage or mild physical damage)’ and ‘severe (severe physical damage requiring intensive treatment)’ categories, taking into account physical impairment and required treatment intensity. The use of alcohol before the attempt means whether the suicide attempter was under intoxication at the moment of suicide attempt and the joint suicide attempts means that there was one or more co-suicide attempters at the attempted suicide.

Two psychiatrists, two psychiatric residents and research assistants reviewed all registered cases once a week at the case discussions. The members of the discussions reviewed the diagnostic validity of each patient. Also, the missing information from the initial assessment was supplemented by additional information obtained after the ED visit.

Ethical approval for the investigation was obtained from the Research Ethics Committee of Yonsei University Wonju College of Medicine, and waiver of documentation of consent has been approved by the committee.

### Statistical analyses

Pearson’s chi - square test was performed to evaluate the difference between socio - demographic factors and suicide - related factors in adolescents and adults. Variables, where expected frequency less than 5 was over 20% were analyzed with Fisher exact test. Bivariate logistic regression analysis was performed with explanatory variables as independent variables to identify risk factors that could predict adolescent suicide attempts compared with adults. Regression model applied Backward, Wald method.

Furthermore, to compare the characteristics of the variables according to sex, men and women were divided and t-test and bivariate logistic regression analysis were performed in the same manner as described above.

All statistical significance was *p* < 0.05 and a two-sided test was used. All analyzes used SPSS version 21.

## Results

### Sociodemographic characteristics (Table 1)

A total of 2003 patients visited the ED after suicide attempt from March 2009 to December 2015. Among them, those older than 65 were excluded from the analysis and a total of 1,576 were the subjects of the study. There were 149 children under the age of 18, and 1,427 people in the age of 19–65. The average age of the two groups was 15.55 ± 1.60, 41.33 ± 11.798 (t = − 76.04, *p* < 0.001).

**Table 1:** Socio-demographic and clinical characteristics of subject

Variable (missing values)	Adolescents (*N* = 149)		Adults (*N* = 1427)		×2	*p*-value
	*N*	%	*N*	%		
Gender						
Male	35	23.5	608	42.6	20.41	< 0.001
Female	114	76.5	819	57.4		
Religion (102)	53	38.4	529	39.6	0.07	0.78
Somatic illness (193)	13	9.6	325	26.1	18.08	< 0.001
Family income (10,000 \) (341)						
0–100	43	36.1	397	35.6	5.51	0.138
101–200	27	22.7	341	30.6		
201–300	23	19.3	211	18.9		
301-	26	21.8	193	15.0		
History of suicide attempts (63)	61	41.5	401	29.4	9.22	0.00
History of psychiatric treatment (159)	53	38.1	508	39.7	0.13	0.71
Family history of suicide (123)	13	9.0	100	7.6	0.34	0.55
Familial psychiatric illness history (112)	14	9.7	159	12.0	0.67	0.41
Psychiatric diagnosis (449)						
Anxiety disorder	0	0.0	4	0.3		1.00
Somatoform disorder	0	0.0	8	0.6	0.84	0.36
Depressive disorder	54	36.2	456	32.0	1.13	0.28
Bipolar disorder	0	0.0	19	1.3	2.00	0.15
Schizophrenia	4	2.7	33	2.3	0.08	0.77
Substance use disorder	2	1.3	164	11.5	14.75	< 0.001
Organic mental disorder	0	0.0	1	0.1		1.00
Adjustment disorder	39	26.2	229	16.0	9.80	0.00
Personality disorder	5	3.4	36	2.5	0.36	0.54

In both groups, there were more women than men, but the rate of women was significantly higher than in adolescents group in the adult group. (76.5% vs 57.4%, × 2 = 20.41, *p* < 0.001).

### Mental health (Table 1)

History of suicide attempt prior to index suicide attempt was statistically more significantly higher than that of adults in adolescents. (41.5% vs 29.4%, × 2 = 9.22, *p* = 0.00). There was no difference between the two groups in the presence of religions, family income, psychiatric treatment, family history of suicide, and family history of mental illness. In psychiatric diagnosis, the adolescents group had fewer substance use disorder and more adjustment disorder than the adult group. (1.3% vs 11.5%, × 2 = 14.75, *p* < 0.001; 26.2% vs 16.0%, × 2 = 9.80, *p* = 0.00, respectively). When analyzing for gender differences, the ratio of adjustment disorders was relatively high (37.1%) in male adolescents than male adults, but in females, the diagnosis of adjustment disorders was not different.

### Suicide-related indicators (Table 2)

Alcohol intake before the suicide attempt was lower in adolescents than in adults. (17.6% vs 64.7%, × 2 = 124.10, *p* < 0.001). 17.6% of adolescents had certain intent to attempted suicide, and 41.1% of adults had certain intent. (× 2 = 22.71, *p* < 0.001). There was no difference between the two groups when comparing pre-planning before the suicide attempt.

**Table 2:** Suicide-related characteristics of subjects

	Adolescents (*N* = 149)		Adults (*N* = 1427)		×2	*p*-value
	*N*	%	*N*	%		
Use of alcohol before the attempt (39)	26	17.6	899	64.7	124.10	< 0.001
Current suicidal ideation (109)						
No	77	56.2	556	41.8	18.86	< 0.001
Yes	44	32.1	399	30.0		
Unknown	16	11.7	375	28.2		
Attempts with suicide plan (245)	25	19.1	195	16.2	0.68	0.40
Intention to die (184)						
Some or little intention	89	82.4	584	58.9	22.71	< 0.001
Certain intention	19	17.6	408	41.1	22.71	< 0.001
Medical lethality (28)						
mild	117	80.1	714	50.9	45.37	< 0.001
moderate to severe	29	19.9	688	49.1	45.37 17.53	< 0.001 < 0.001
Joint suicide attempts (215)	7	5.3	11	0.9		
Admission (299)	35	23.5	455	31.9	4.43	0.03
Motivation of the suicide						
Psychiatric (127)	27	24.7	323	24.7	2.13	0.14
Interpersonal (85)	115	79.9	860	63.8	14.74	< 0.001
Job-related (126)	21	14.6	252	19.3	1.88	0.17
Economic (111)	6	4.3	344	26.0	33.08	< 0.001
Illness-related (123)	2	1.4	86	6.6	5.97	0.01
Loss of family member (123)	3	2.1	34	2.6	0.11	0.73
Abuse-related (125)	8	5.6	14	1.1	17.87	< 0.001
Legal problem (129)	0	0.0	8	0.6	0.86	0.35
Method of suicide attempt						
Poisoning	101	67.8	946	66.3	0.13	0.71
Analgesics	43	28.9	56	3.9	142.48	< 0.001
Psychiatric medication	28	18.8	441	30.9	9.46	0.00
Pesticide and Herbicide	5	3.4	293	20.5	25.9	< 0.001
Other medications	27	18.1	266	18.6	0.02	0.87
Asphyxia by gas	9	6.0	152	10.7	3.12	0.07
Hanging	5	3.4	106	7.4	3.41	0.06
Injury by sharp object	26	17.4	171	12.0	3.68	0.05
Jumping and Drowning	6	4.0	34	2.4	1.47	0.22
Others	2	1.3	18	1.3	0.00	0.93

In medical lethality, adolescents were mostly mild, with 80.1%. However, Adults tended to attempt suicide in a relatively more lethal manner, which moderate to severe (49.1%) accounting for almost half of the cases. (× 2 = 45.37, *p* < 0.001) 5.3% of adolescents attempted joint suicide, significantly more than 0.9% of adults (× 2 = 17.53, *p* < 0.001). This tendency was statistically significant for women only (6.9% vs 0.9%, × 2 = 20.24, *p* < 0.001). The rate of admission after the suicide attempt was statistically lower than 31.9% in adults with 23.5% in adolescents. (23.5% vs 31.9%, × 2 = 4.43, *p* = 0.03).

As a motive for suicide, adolescents were more likely to attempt suicide due to interpersonal problem and abusive issues than adults. (79.9% vs 63.8%, × 2 = 14.74, *p* < 0.001; 5.6% vs 1.1%, × 2 = 17.87, *p* < 0.001, respectively). On the other hand, Motivations due to economic causes (4.3% vs 26.0%, × 2 = 33.08, *p* < 0.001) and illness-related causes (1.4% vs 6.6%, × 2 = 5.97, *p* < 0.01) were lower than adults.

In method of suicide attempt, adolescents used more ways of taking analgesics than adults. (28.9% vs 3.9%, × 2 = 142.48, *p* < 0.001) Psychiatric medications poisoning (30.9% vs 18.8%, × 2 = 9.46, *p* = 0.002) and pesticide and herbicide poisoning (20.5% vs 3.4%, × 2 = 25.96, *p* < 0.001) were less than adults.

### Binary logistic regression analysis for factors associated to suicide attempt (Table 3)

The adjusted Odds ratio obtained by performing binary logistic regression analysis is shown In Table 3. After adjustment, the factors that have a significant difference between adolescents and adults were the presence of physical illnesses, the history of previous suicide, the alcohol intake prior to suicide attempts, intention to die, lethality of suicide attempts, economic motivation, analgesics poisoning, psychiatric medications poisoning, pesticide and herbicide poisoning, and substance use disorder.

**Table 3:** Binary logistic regression model of factors associated to suicide attempt

Variable	OR (95% CI)	*P*	aOR (95% CI)	*P*
Gender	0.41 (0.27–0.61)	< 0.001		
Somatic illness	0.30 (0.16–0.53)	< 0.001	0.38 (0.17–0.82)	0.01
History of suicide attempts	1.70 (1.20–2.41)	0.00	2.85 (1.63–4.97)	< 0.001
Use of alcohol before the attempt	0.11 (0.07–0.18)	< 0.001	0.09 (0.05–0.17)	< 0.001
Current suicidal ideation				
No		< 0.001		
Yes	0.79 (0.53–1.17)	0.25		
Unknown	0.30 (0.17–0.53)	< 0.001		
Intention to die				
Some or little intention				
Certain intention	0.30 (0.18–0.50)	< 0.001	0.39 (0.19–0.80)	0.01
Medical lethality				
mild				
moderate to severe	0.25 (0.16–0.39)	< 0.001	0.458 (0.24–0.86)	0.01
Joint suicide attempts	6.14 (2.34–16.13)	< 0.001		
Motivation of the suicide				
Interpersonal	2.24 (1.47–3.42)	< 0.001		
Economic	0.12 (0.05–0.28)	< 0.001	0.19 (0.07–0.51)	0.00
Illness-related	0.20 (0.05–0.836)	0.02		
Abuse-related	5.52 (2.27–13.40)	< 0.001		
Method of suicide attempt				
Analgesics	9.93 (6.37–15.48)	< 0.001	5.00 (2.30–10.87)	< 0.001
Psychiatric medications	0.51 (0.33–0.79)	0.00	0.40 (0.21–0.75)	0.00
Pesticide and Herbicide	0.13 (0.05–0.33)	< 0.001	0.11 (0.02–0.49)	0.00
Psychiatric diagnosis				
Substance use disorder	0.10 (0.02–0.42)	0.00	0.14 (0.01–1.18)	0.07
Adjustment disorder	1.85 (1.25–2.74)	0.00		
Admission at medical institutions	0.65 (0.44–0.97)	0.03		

Compared to adults, the OR of past suicide attempt was 2.85 times higher after the correction, and the proportion of alcohol intake was 0.09 times lower. The adjusted OR of intention to die was 0.39 (95% CI 0.19–0.80, *p* = 0.01), the adjusted OR of medical lethality above moderate was 0.45 (95% CI 0.24–0.86, *p* = 0.01).

In adolescents, the OR of suicide attempts with analgesics as compared to adults was 5.00 (95% CI 2.30–10.87, *p* < 0.001). In contrast, psychiatric medication was 0.40 (95% CI 0.21–0.75, *p* = 0.00) and pesticide and herbicide poisoning was 0.13 (95% CI 0.02–0.49, *p* = 0.00).

For the psychiatric diagnosis, after correction, the adjustment disorder had no significant effect on the difference between two groups. But, in adolescents, the OR of the substance use disorder was found to be much smaller by 0.14 times. (95% CI 0.01–1.18, *p* = 0.07).

## Discussion

This is the first study to directly compare the characteristics of suicide attempts in adolescents who visit the ED compared to those of adults. We tried to discover the characteristics of suicide attempts in adolescents, comparing social demographic, clinical, suicide-related factors, for a total of 149 adolescents and 1427 adults under the age of 65.

Women in both groups were more than men, which was the same as the results of previous studies [11, 18, 19]. The rate of hospitalization was significantly higher in adults. (23.5% vs 31.9%). Previous studies have shown a various rate of hospital admissions after suicide attempts. In one study, about 34% of adolescent suicides attempters were hospitalized [15], and in another study, 26.9% of attempters were hospitalized [20]. In addition, about one-third of the patients visiting the ED was hospitalized after attempting regardless of age [19]. Admission rates are influenced by various factors, such as the medical system and management guideline, so it is difficult to directly compare them. In this study, it is estimated that the high medical lethality of suicides in adults is associated with the high admission rates in adults.

At the time of visiting the ED, 41.5% of the adolescents had a history of suicide attempt, which was significantly higher than adults. The rate of suicide attempts in previous adolescent suicide attempts in the ED was 53.3% in the UK and 37.9% in the European multicenter study, similar to those in the present study [21, 22]. In previous studies compared with adults, one study found that adults were more likely to attempt suicide [8], but, other study reported no significant difference [11]. These studies were conducted on inpatients, so the proportion of patients who had a history of attempt is high and difficult to compare with the results of this study. Also, in this study, since adolescents tend to attempt suicide with relatively little intent compared to adults, these pattern of suicide attempts may have been repeated. However, as in adults, adolescents’ repeated suicide attempts increase the risk of subsequent suicide re-attempts and are also a factor that increases the likelihood of suicide death [23]. According to previous studies, about 15% of suicide re-attempts occurred in six months, 13.6–24% in a year [24–26]. In addition, considering our findings shows that adolescents had little previous psychiatric diagnosis or history of treatment, in reality, psychological assessment and intervention after suicide attempt may not have been properly made. This suggests that it is urgent and important to establish strategies for early detection and intervention after the first suicide attempt by adolescents.

In the adolescents group, diagnosis of an adjustment disorder was greater than that of adults, and it was consistent with the results of previous comparison study [8]. Also, 21% of adolescents in psychological autopsy study conducted in Finland were diagnosed as adjustment disorders, similar to 26% in this study [27]. However, the difference was significant only in male adolescents. One study showed that female adolescents report more of the symptoms of depressed mood and guilt than male in depressive disorder [28]. It cannot be ruled out that the female adolescents were more likely to have been diagnosed with depressive disorder due to the high level of expression of symptoms, whereas the male adolescents were diagnosed with an adjustment disorder, in the ER where diagnosis is made with limited information. Research using a structured interview tools on more samples should be carried out to identify gender differences in the psychiatric diagnosis of adolescents.

The results of our study show that a majority of adolescents attempt suicide in the context of interpersonal conflicts, whereas a majority of adults attempt suicide in the context of economic problems. These findings are consistent with the earlier studies that have reported interpersonal issues as the most common motivation for adolescents’ suicide attempts [7, 11, 18, 21]. Various changes in relationships, emotional instability, and a lack of coping skills in dealing with them may lead adolescents to suicide attempt [7, 29]. Therefore, it is necessary to provide educations on coping skills for various interpersonal issues and stress management to prevent adolescents’ suicide attempt.

Poisoning was the most common method of suicide attempt for both groups. But adults were more likely to try to suicide attempt by overdosing on psychiatric medications or herbicides/pesticides while adolescents were more prone to analgesics. It is consistent with previous studies that adolescents prefer to take analgesics as a way to suicide attempt [8], and there are also reports that legislation regarding package size of analgesics is effective in preventing suicides [30]. This is understandable in terms of availability, as analgesics are easy to get. Another consideration is the high risk of suicide in adolescents already abusing analgesics. The previous study has shown that approximately 4.7% of adolescent misuse over-the-counter drugs, with higher prevalence of psychiatric illness and suicides than non-users [26]. Because some adolescents who abuse analgesics use them to reduce sleep or anxiety [31], it is possible that the adolescents who used the analgesic drug as a self-medication used it as a way to suicide attempt. This can be seen as similar to using psychiatric drugs in adult suicide attempters. Assessment of risk of suicide might be required in adolescent patients suspected of abuse of analgesics, as well as evaluation of analgesics abuse is needed in adolescent at suicide-risk.

A notable result was that adolescents had more attempted joint suicide than adults. In particular, the difference was significant only among female adolescents. Previous research reported that contagious nature of self-harm appears in young adults, especially in female adolescents [32]. Contagion in suicidal behavior has been explained as being caused by assortative selection of peer group or shared relationship stress [32, 33]. In our study, by similar mechanisms, it is estimated that female adolescents’ joint suicides were reported more commonly. Female has a higher empathy for others than male [34]. So, we can consider the possibility that the motive was enhanced by others to attempt joint suicide or the suicidality was shared by vulnerable individuals in stressful state [33]. Furthermore, considering that most suicide attempters communicate their suicidal intentions prior to suicide, we should pay attention to suicidal communication before suicide [35]. Previous study had limits to identify the contagious nature of suicides at the school level. The results of this study provide more direct evidence to the hypothesis that adolescent suicides tend to be more contagious, since the results show that adolescents try to joint suicide attempt more than adults. However, researches on joint suicide are scarce, especially in studies aimed at adolescents. The study was conducted on patients visiting the ED only, so it was not clear whether adolescents attempted joint suicide more than adults. Although there is no significant difference between both groups, it is possible that the proportion of transfer to the ED after being rescued is relatively small because adults attempt suicide in a fatal method such as CO intoxication. Therefore, a study on the joint suicides of adolescents, especially of female, is needed in the future.

### Strengths and limitations

This study has the benefit from a relatively large sample based on data collected from patients visiting the ED. Our study could supplement the limitations of representation in previous studies of inpatients.

However, this feature also acts as a limitation. Because patients are evaluated based on ED, it is often difficult to collect information with the patient at unconscious mental state or confusion, or with uncooperative patients. As a result, the major limitation is that the missing values are relatively high. To compensate for this, the same analysis was carried out with the complete date set and it was confirmed that there was not much difference in overall tendency. In addition, since it is a sample that has only been recruited from one hospital, a lack of demographic representation is also a limitation. Finally, because the analyses focused on adolescents versus one adult group, not different adult populations by age, the results have some limits, as it is difficult to identify the findings that were being influenced by a particular age group; thus, future studies will require more elaborate age-specific comparisons.

## Conclusions

This study is the first direct comparison study of the socio-demographic, clinical, and suicide-related factors between adolescents and adults based on ED. Interpersonal problems are the main motivation for adolescent suicides, and previous suicide attempts were more frequent than adults. It also showed a tendency to attempt suicide with a lower intention to die and a relatively lesser degree of lethality than adults. Among them, taking analgesics is the most common method, so measures need to be taken against over-the-counter drug use by adolescents. Major depressive disorders were also the most common diagnosis, with adjustment disorders accounting for a significant proportion. Therefore, based on these results, there is an early need for therapeutic and social intervention to prevent adolescent suicide attempts. Our results highlight the importance of specific and individualized preventive strategies for adolescent suicide attempters. Moreover, careful assessment and appropriate treatment for underlying psychopathologies in suicide attempters among adolescents should be considered.

## Data Availability

The datasets used and analyzed during the current study are available from the corresponding author on reasonable request.

## Abbreviations

DSM-IV-TR: Diagnostic and Statistical Manual of Mental Disorders-IV-Text Revised
ED: Emergency Department
OECD: Organization for Economic Cooperation and Development
OR: Odds Ratio
WHO: World Health Organization
